# A method for detecting epistasis in genome-wide studies using case-control multi-locus association analysis

**DOI:** 10.1186/1471-2164-9-360

**Published:** 2008-07-31

**Authors:** Javier Gayán, Antonio González-Pérez, Fernando Bermudo, María Eugenia Sáez, Jose Luis Royo, Antonio Quintas, Jose Jorge Galan, Francisco Jesús Morón, Reposo Ramirez-Lorca, Luis Miguel Real, Agustín Ruiz

**Affiliations:** 1 Neocodex, Avda. Charles Darwin 6, Acc. A, 41092 Sevilla, Spain; 2 Wellcome Trust Centre for Human Genetics, University of Oxford, Oxford OX3 7BN, UK

## Abstract

**Background:**

The difficulty in elucidating the genetic basis of complex diseases roots in the many factors that can affect the development of a disease. Some of these genetic effects may interact in complex ways, proving undetectable by current single-locus methodology.

**Results:**

We have developed an analysis tool called Hypothesis Free Clinical Cloning (HFCC) to search for genome-wide epistasis in a case-control design. HFCC combines a relatively fast computing algorithm for genome-wide epistasis detection, with the flexibility to test a variety of different epistatic models in multi-locus combinations. HFCC has good power to detect multi-locus interactions simulated under a variety of genetic models and noise conditions. Most importantly, HFCC can accomplish exhaustive genome-wide epistasis search with large datasets as demonstrated with a 400,000 SNP set typed on a cohort of Parkinson's disease patients and controls.

**Conclusion:**

With the current availability of genetic studies with large numbers of individuals and genetic markers, HFCC can have a great impact in the identification of epistatic effects that escape the standard single-locus association analyses.

## Background

Most common diseases have an etiology so complex that years of research have yielded scarce results towards the elucidation of their causes. Technology and methodology are improving quickly but results have been arriving slowly. Nonetheless, optimism is in the air, because large studies of many individuals and genetic markers seem to finally be revealing some of the genetic factors behind these common diseases [1].

The difficulty of elucidating the genetic basis of complex diseases roots in the many factors that can affect the development of a disease. Many factors, both genetic and environmental, each with possibly only a small effect, may be necessary for the expression of a particular disease phenotype. For example, most associations reported in the recent wave of genome-wide association studies of different common diseases exhibited small (1.1–1.4) to moderate (1.5–2) odds ratios [2].

These small effects may only be detectable by means of genetic association analysis in very large samples, or in smaller sub-samples in which, by sample selection, this effect is enlarged: a sub-sample where the allele frequency of a particular risk gene is increased; or a sub-sample where a combination of other alleles or environmental factors act to increase the observable effect of a particular gene [3].

Many genes may contribute to the expression of complex diseases. It is quite reasonable to expect that the effects of some of these genes do not sum up in a simple fashion. Epistasis generally refers to an interaction between the effects of genes at different loci, although the term has been used in different contexts by different disciplines [4]. Some of these genetic effects may interact among them, such that the presence of two or more particular genes may increase the risk to a disease more than expected from their independent effects, the expectation being derived from a pre-defined model, such as additive or multiplicative. For example, the odds ratio for an epistatic effect of two genes may be larger, even much larger, than the combined effect (sum or product) of each of the two single genes [5,6]. Moreover, there are biological models of epistasis where genes only have epistatic effects [7], such as a two-locus mutation masking a known phenotype. Some of these genetic effects may prove undetectable by current single-locus methodology [8]. There have been some early attempts to search for epistatic effects [5,9-11], but there is currently a need for methods to study this important genetic phenomenon, perhaps key for complex diseases.

A wealth of current research in molecular genetics has discovered millions of genetic markers which provide a good coverage of common genetic variation across the entire human genome [12]. At the same time, advances in genotyping technology have greatly increased the quantity and quality of genotypes. Current genotyping platforms can generate millions of genotypes in short periods of time. These events have made possible the genetic association analysis of a trait across the entire genome.

Although the arrival of genome-wide association testing is great news for the genetic dissection of complex traits, the large number of statistical tests involved raises the issue of statistical significance. For example, to maintain a Type I error of 5 percent when testing 100,000 markers for genetic association may require a test-statistic with a probability value of 5 × 10^-7^, if a Bonferroni correction is applied. Nonetheless, many of these markers are correlated so this correction would be too strict, but in any case the required p-value would be very small.

This problem of multiple testing is even more extreme for the test of epistasis. For example, for 100,000 markers, there are a total of 5 × 10^+9 ^two-locus combinations, which would require a Bonferroni-corrected p-value of 1 × 10^-11 ^for a genome-wide significance level of 0.05, which again would be overly conservative due to the correlated nature of many of these tests. To achieve these significance levels it is necessary to study large samples and expect to find large epistatic effects.

Replication of findings in independent samples is sought for growing confidence in statistical results. The lack of replication of association results may be due to different causes, some technical (low power due to small samples, bad quality of phenotypic or genotypic data, uncorrected noise or covariates) and some biological (heterogeneity of effects or population-specific risks). An approach to tackle the multiple testing issue is to divide the available sample into independent groups and to carry out the analysis in these independent groups to look for consistent results across the groups. Some true genetic effects will be missed due to lack of power (due to the reduced sample in each group) and to heterogeneity, but this approach may allow the identification of moderate/large-sized epistatic effects that are frequent and consistent.

In this scenario, we have developed an analysis tool to search for genome-wide epistasis in a case-control design. Hypothesis Free Clinical Cloning (HFCC) is an standalone software which allows for single-locus genetic association testing, as well as epistasis testing for multi-locus combinations of markers. Due to the intense computational burden, it is programmed to take advantage of computer clusters by dividing the tasks into processes which can migrate to the available CPUs. We present here the method, as well as a genome-wide two-locus epistatic analysis performed on a real dataset of Parkinson's disease that illustrates the method. With the current availability of genetic studies with large numbers of individuals and genetic markers, HFCC can have a great impact in the identification of epistatic effects that escape the standard single-locus association analyses.

## Methods

### Input Data

#### Sample

The standard input to HFCC is a case-control sample with hundreds or thousands of individuals. Similarly to other association methods, it is convenient that cases and controls are matched for potentially important covariates like age, sex, ethnicity, geographical location, environmental factors, etc. Dichotomous phenotypes with a potential effect on the trait may be used as covariates in the analysis.

If all cases have only one disease phenotype or trait, HFCC carries out a single-phenotype analysis. In the single-phenotype scenario, HFCC can analyze the full sample simultaneously for extra statistical power. In addition, we have developed a multi-group analysis strategy, explained in more detail in a later section, that allows the replication of consistent results, and it also aids the elimination of false positives results, a very attractive quality for genome-wide analysis of large number of genetic markers. For this single-phenotype multi-group analysis option, cases can be sub-divided into groups to allow for replication of consistent results across these groups. Controls can also be sub-divided into groups to eliminate spurious associations.

Nonetheless, one of the strengths of HFCC is that it can analyze multiple phenotypes simultaneously. For this multiple-trait analysis, several groups of cases with different but related phenotypes are formed, each matched to its own control group. Indeed, the multi-group analysis strategy is especially convenient for the simultaneous analysis of several related diseases, or different symptoms of a syndrome, so that we can identify the genetic effects common to the different groups.

#### Genotypes

HFCC can currently analyze di-allelic markers such as single nucleotide polymorphisms (SNP), and it can also accommodate other dichotomous markers such as the presence or absence of a particular allele of a multi-allelic marker or of a haplotype. HFCC can analyze anything from small sets of candidate gene markers to genome-wide arrays of hundreds of thousands of markers.

Markers can be filtered out before analysis if they have low call rate, low minor allele frequency, or if they fail a Hardy-Weinberg equilibrium test. Nonetheless, data analysis filters inherent in the software can eliminate, at least partly, these problematic markers.

Currently, linkage disequilibrium (LD) among markers is ignored during the analysis, although it is useful for the validation and interpretation of results.

#### Datasets

To illustrate the method we used data from the SNP Resource at the NINDS Human Genetics Resource Center DNA and Cell Line Repository . The original genotyping was performed in the laboratory of Drs. Singleton and Hardy (NIA, LNG), Bethesda, MD USA [13]. We have used data on 270 patients with Parkinson's disease and 271 normal control individuals who were genotyped for 396,591 SNPs in all 22 autosomal chromosomes using the Illumina Infinium I and Infinium II assays. Cases were all unrelated white individuals with idiopathic Parkinson's disease and age of onset between 55–84 years (except for 3 young-onset individuals). The control sample was composed of neurologically normal, unrelated, white individuals from the USA.

To explore the power of HFCC we also analyzed a simulated dataset that was originally generated to evaluate the power of a different gene-gene interaction method [14]. These case-control data were simulated under different genetic models, and different sources of noise (genotyping error, missing data, phenocopy and genetic heterogeneity). For each genetic model and noise condition, 100 datasets were generated. Each dataset contains 200 cases and 200 controls, with genotypes for 10 SNPs under HWE. An epistatic effect with no single-locus marginal effect was simulated for a pair of SNPs, and the remaining 8 SNPs were simulated under the null hypothesis of no genetic effect. For the genetic heterogeneity case, two epistatic effects (each due to a different pair of SNPs) were simulated. More detail of these simulated datasets can be found in the original publication [14].

### HFCC Modelling

#### Statistical tests

##### Single-Locus Association tests

HFCC can perform a single-locus genome-wide association scan. A di-allelic marker with alleles A and B, has 3 possible genotypes: AA, AB and BB. For each genetic marker, HFCC performs three statistical tests, comparing each genotype against the other two. For example, the frequency of the AA genotype is compared against the combined frequency of the AB-BB genotypes, in cases and controls. This could be considered a dominance model test for the B allele. Similarly, recessive (BB versus AA-AB) and heterozygote models (AB versus AA-BB) are also considered. The test for association between the established genotypic classes and the case-control groups can be a Wald test or a chi-squared test with one degree of freedom (df).

##### Multi-Locus and Epistatic Association tests

HFCC can also perform a multi-locus genome-wide association scan. For a two-locus scan, HFCC first forms all possible combinations of two markers from all available markers. For each two-marker combination (marker 1 with alleles A and B, and marker 2 with alleles C and D), there are 9 possible genotypic classes (AACC, AACD, AADD, ABCC, ...., BBDD), and a total of 512 fully penetrant disease models [7]. For our purpose, this number of models can be reduced to 255, because some models are redundant or represent a model with no genetic effect. These 255 models include a variety of standard models (single-locus, double-recessive, double-heterozygote, etc.) as well as other rare models. The user can select to test all available models or a subset of them.

As in the single-locus case, the statistical test involves comparing the frequency of two sets of genotypic classes in cases versus controls. The two sets of genotypic classes are defined by the model being tested. For example, in the case of the double-recessive model, the frequency of the BBDD genotypic class is compared against the combined frequency of all other genotypes (AACC through BBCD), in cases and controls, from which a Wald-test Z statistic, or a chi-squared statistic with one degree of freedom, can be obtained.

Another analysis option is a more general multi-locus test [6] which compares two chi-squared statistics with four degrees of freedom (one obtained for cases and the other one for controls). This test, although more general, may not be able to detect some disease models.

For a three-locus scan, there are 27 possible genotypic classes, and the software is currently implemented to test the 27 simplest 3-locus genetic models, comparing the frequency of a genotypic class (ie, AACCEE) against the combined frequency of all other genotypes.

##### Post-Hoc tests

A variety of post-hoc statistical tests are included in the post-hoc analysis software, named Alambique. For single-locus analysis, it is possible to carry out Hardy-Weinberg equilibrium, heterozygous, homozygous, allele positivity (dominance), recessive, common odds ratio (Armitage's trend) [15], and genotypic (2 degrees of freedom) tests.

For the two-locus analysis, all 255 fully-penetrant models can be tested. Moreover, interaction indices can be estimated to determine to which extent an observed two-locus association deviates from an additive model [16]. Finally, departures from a multiplicative model can be tested using a case-only chi-squared test [17].

These post-hoc analyses are useful to determine which genetic model explains better an observed association, and to measure the epistatic component of associated multi-locus combinations.

#### Grouping

##### Replication (Case versus control) groups

The statistical tests for association of a trait with a marker are based on the differential frequency between cases and controls of a particular allelic or genotypic combination. Comparing the full sample of cases versus the full sample of controls is the most powerful approach to find statistically significant associations. Nonetheless, to evaluate genomewide association or epistasis we need to test a very large number of markers or marker combinations, which in turn can produce a large number of spurious results. Therefore, we need to improve the filtering of false-positive results, at the expense of increasing the false-negative findings.

We have approached this multiple-testing issue by partitioning the available sample into multiple replication groups. Cases are selected randomly to be part of one and only one case group, and similarly for control individuals and control groups. Then, the statistical analysis is performed on each of the paired case-control groups, and results that are consistent across all replication groups are selected. This sample-splitting technique may prove less powerful to detect positive results in general, but provides a powerful tool to eliminate false positives, thereby highlighting potentially true effects that are consistent across samples. The number of groups into which the sample is partitioned can be chosen by the user depending on the study design, and the number of subjects and markers available for analysis.

This multi-group strategy is one of the differentiating aspects between HFCC and conventional association methods, and it is particularly useful for the analysis of multiple related phenotypes. Case groups are directly defined by phenotypic criteria, and control groups are matched to each case group. This type of analysis can reveal genetic associations that are common to these phenotypes, revealing a common etiology for multiple symptoms of a disease, or for several related diseases [18].

##### Control filter (Control versus control) groups

HFCC has developed an efficient noise filter, by applying the association tests to independent groups of controls. Positive results that arise from a comparison of two groups of controls must be spurious associations due to marker or sample characteristics. These spurious associations can be filtered out of the results from the case-control analysis, providing an efficient sample-specific background noise filter.

### Algorithm and program execution

**Figure 1:**
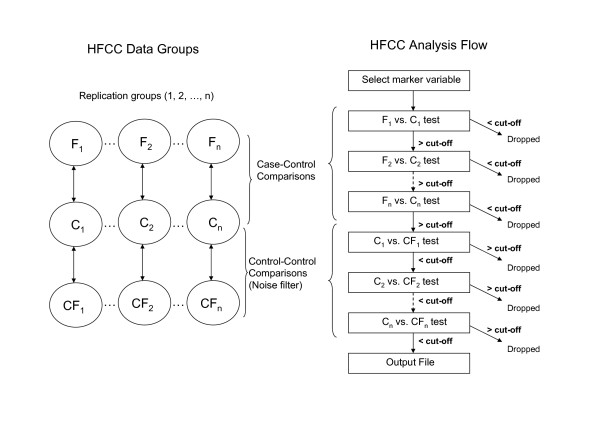
**HFCC data groups and algorithm**. HFCC data groups are divided into cases (F), controls (C) and control filter (CF). The number of replication groups is experiment-specific and depends on the available dataset (sample size and number of genetic markers). The HFCC algorithm is defined by a particular analysis flow. For each marker variable (single-locus markers, two-locus marker combinations, etc. depending on the type of study), a sequential number of tests is performed. Case-controls comparisons are performed on each replication group, and control-control comparisons are carried out in each control filter group. If any of these tests is not beyond a statistical threshold, the marker variable is dropped, and the next marker variable is analyzed. Marker variables over the statistical threshold in all case-control tests, and below the threshold in all control-control tests, are selected.

HFCC requires specific data and parameter files. The input data file is a simple-text matrix of integers in which rows represent genetic markers and columns represent individuals. Each integer represents the genotype for one individual at one marker, coded as 0 for missing, 1 for one homozygote, 2 for the heterozygote, and 3 for the other homozygote. These integers are entered sequentially, with no blank spaces or lines, to save space. There is one data matrix for each group of cases (F1.txt, F2.txt, etc.), controls (C1.txt, C2.txt, etc) and noise-filter controls (CF1.txt, CF2.txt, etc.), as can be seen in Figure 1. A parameter data file defines the type of analysis (single- or multi-locus; genetic model; test statistic), number of genetic markers, number of groups, sample size per group, statistical cut-off for significance, and other necessary parameters.

The following algorithm describes HFCC analysis flow (Figure 1). Genetic markers are analyzed sequentially in the order in which they are entered in the data matrices. First, HFCC selects a marker variable (either a single-locus marker or a multi-locus combination of markers, depending on the type of analysis). Then, for the selected analysis model, it computes genotype frequencies and test statistics in each replication group, sequentially. If the test statistic is smaller than a pre-defined cut-off in any of the replication groups, the marker variable is dropped at that stage and no more computations are performed on this marker variable to save processing time. If the test statistic is larger than the cut-off in all replication groups, then the marker variable is written to the output file and also entered in the control noise filter. This noise reduction analysis computes frequencies and test statistics in each control filter (control versus control) group sequentially. If the test statistic is larger than a pre-defined cut-off in any of the filter groups, the marker variable is flagged for removal from the final output file. The marker can fail at any of the sequential group analyses (replication or control filter group comparisons), at which point that marker is dropped to save processing time. This procedure is repeated for all possible marker variables, writing to the output file all associations considered statistically significant. This output file contains the marker (or marker combination) and the genetic model for which it yielded a positive association (ie, for a two-locus combination: marker 1, genotypic class 1, marker 2, genotypic class 2).

These selected marker variables can then be included in two sequential post-hoc analyses which aid in the interpretation of the results. The first post-hoc analysis yields odds ratios and chi-squared values for all selected marker variables. It also applies a direction filter, by selecting only those results which display the same direction of effect in all replication groups, and writes a new output file, which contains only those marker variables with a significant and consistent effect (same direction and same model) in all replication groups.

The second post-hoc analysis (Alambique) identifies the best type of genetic model for each association selected in the first post-hoc analysis. For this reason, all replication groups are combined into a single group, that is, all cases in one case group and all controls in a control group. Alambique has its own noise elimination algorithm, denominated tracking filter. Many of the selected associations are due to what we call a tracking marker, a locus with a very large marginal effect. Many of these tracking markers are not in HWE and are filtered at this stage. In any case, having a large marginal effect makes a locus appear in many two-locus associations, because the large effect tracks other loci. Because our focus is on finding epistatic interactions, the many positive associations due to these tracking effects can be filtered out and analyzed independently. For the remaining marker variables, which have passed this tracking filter, there is one analysis option that groups the two-locus results into those with marginal effects for only one locus (conditional effect), or for both loci (simultaneous effect) or those without marginal effects (epistatic effect) [19]. Some of the conditional and simultaneous results may still exhibit epistatic effects if the two-locus effect size deviates from an additive or multiplicative expectation, and these epistatic effects can also be flagged. A second analysis option tests all 255 fully-penetrant models in selected marker variables, to help choosing the model that best fit the data.

Due to the extremely time-consuming and computer-intensive nature of these analyses, especially for genomewide multi-locus analysis, it will often be necessary to run HFCC under a small selection of genetic models, and then use Alambique to identify the best genetic model for the selected markers. The number and selection of genetic models to analyze will depend on the phenotype, the dataset, previous knowledge and computer resources available.

## Results

We have applied HFCC to both simulated and real datasets to illustrate the power and the functioning of the method.

### Simulated data analysis

To evaluate the power of the method, we analyzed a simulated dataset published previously [14]. Data were simulated under three types of two-locus epistasis models: Model 1 was a logical XOR model, a combination of exactly one heterozygous and one homozygous loci (i.e., for two markers with alleles A, B and C, D respectively, risk genotypes would be ABCC, ABDD, AACD and BBCD), similar to M170 [7] but with variable penetrances; Model 2 involved the inheritance of exactly two, and only two, risk alleles (i.e., A and C) from any of two different loci (i.e., risk genotypes would be AADD, ABCD, and BBCC), such as M84 [7]; Model 3 was a variable penetrance risk model with a protective double-heterozygote (i.e., protective genotype would be ABCD), similar to M16 [7]. In addition, to make the simulated data more similar to real data, and therefore to evaluate the effect of noise in the detection of these epistatic effects, different sources of noise were modeled in the simulations. For each of the three types of genetic models described above, the data was simulated and analyzed under six types of noise: without noise, with 5% genotyping error (GE), with 5% missing data (MS), with 50% phenocopy (PC), with 50% genetic heterogeneity (GH), and with all sources of noise simultaneously. For more information on these simulated datasets please refer to the original publication [14]. Power was defined as the proportion of times the correct model was detected out of each set of 100 simulations. For genetic heterogeneity the correct model was defined as detection of either one of the two epistatic loci simulated.

To detect potential epistatic loci, we ran HFCC two-locus analysis with nine simple genetic models, the M1, M2 and M16 models [7]. There are a total of 45 possible two-locus combinations that can be formed with the 10 simulated genetic markers. Due to this relatively small number of tests (relative to a genome-wide study), HFCC was performed with only one group of cases and controls, and a chi-square cut-off of 6.64 (approximately a p-value = 0.01 with 1 degree of freedom). Using only one case-control group allows also for direct comparison of HFCC's power to the power estimates published previously for this dataset. Results from the SNPs simulated under the null hypothesis reveal fewer Type I errors than expected, confirming that the method is not biased and perhaps conservative. This reduced number of false positives may reflect that some of the epistasis tests used were correlated, as well as a general lack of power to detect some epistatic effects, especially for rare genotypes.

HFCC had excellent power (>96%), with a Type I error of 0.01, to detect the simulated two-locus interactions under most genetic models and noise conditions, including genotyping error and missing data (See Table 1). Genetic heterogeneity reduced the power to 82% under model 3, and the presence of phenocopies had also a significant impact on power for models 1, and especially, 3. Finally, if all four sources of noise were acting simultaneously the power was reduced to 51% for model 1, 71% for model 2, and 34% to model 3.

**Table 1:** Power Analysis.

	Power (%)		
Noise	Model 1	Model 2	Model 3
None	100	100	99
GE	100	100	96
MS	100	100	97
PC	89	100	49
GH	100	100	82
GE+GH+PC+MS	51	71	34

Power of HFCC to detect a two-locus interaction at an alpha level of 0.01 under 3 genetic epistasis models and different sources of noise: no noise (None), genotyping error (GE), missing data (MS), phenocopy (PC), genetic heterogeneity (GH), and all sources of noise simultenously (GE+GH+PC+MS). For the datasets with genetic heterogeneity, we reported the power to detect either of the two simulated two-locus effects. Model 1 is a heterozygote-homozygote risk (i.e., risk genotypes ABCC, ABDD, AACD and BBCD); Model 2 represents a "2 and only 2"-allele risk (i.e., A and C) from any of two loci (i.e., risk genotypes AADD, ABCD, and BBCC); Model 3 represents a protective double-heterozygote (i.e., protective genotype ABCD).

### Parkinson's disease analysis

To demonstrate the HFCC software in real data we analyzed the open-access Parkinson's dataset described in the Methods section. We included all 396,591 SNP markers. Many of them had low minor allele frequencies or failed Hardy-Weinberg equilibrium (HWE), but were included in order to test the data filters inherent in HFCC, which are meant to eliminate, at least partially, these problematic markers.

The 270 cases and 271 controls were separated into groups, to illustrate HFCC multi-group analysis strategy. We created 3 replication groups of 90 cases each (groups F in Figure 1) and 3 groups of controls of approximately similar size (C in Figure 1; one group with 91 individuals and two groups of 90 individuals). Because of the lack of more control individuals, the noise filter control groups (CF in Figure 1) were selected respectively from each of the control groups, chosen so that the noise filter would not pair two identical control groups (ie, CF1 = C3; CF2 = C1; and CF3 = C2). Ideally, independent groups of C and CF controls would be used, so we expected that the noise filter in this experiment, in which the same groups of controls were used as C and CF, would not be as effective.

To detect potential epistatic loci, we ran HFCC two-locus analysis with nine simple genetic models, the M1, M2 and M16 models [7]. These nine genetic models were tested in all possible (78.6 × 10^+9^) two-locus marker combinations. In order to be considered a preliminary positive result, the chi-squared (1 df) cut-off value was set at 6.64, which yields a probability value of 0.01 for each replication group (p < 10^-6 ^over all three replication groups). Although this p-value may be considered low compared to the number of statistical tests performed, this is only a cut-off to select preliminary positive results, which are then filtered and subjected to post-hoc analysis to select the most promising results. We obtained a total of 418,535 preliminary two-locus associations at this p-value cut-off (Table 2). To evaluate the impact of the different noise filters (control, direction and tracking) we applied them selectively to these preliminary results.

**Table 2:** HFCC analysis: Preliminary results and effect of noise filters.

Applied Filters	Unfiltered Results	After Control Filter	After Direction Filter	After Tracking Filter	Selected two-locus SNP pairs		
					Simultaneous	Conditional	Epistatic
Method I							
N	418,535	-	320,265	-	71,332	248,898	35
%	100%	-	76.5%	-	17.0%	59.5%	0.01%
Method II							
N	418,535	-	320,265	26,371	19,630	6,706	35
%	100.0%	-	76.5%	6.3%	6.1%	2.1%	0.01%
Method III							
N	418,535	340,043	320,188	26,347	19,611	6,701	35
%	100.0%	81.2%	76.5%	6.3%	6.1%	2.1%	0.01%

Method I: Only Direction Filter applied.

Method II: Direction and Tracking filters applied.

Method III: All filters applied (Control, Direction, and Tracking filters sequentially in that order).

N: Number of selected two-locus SNP pairs.

%: Percentage of selected two-locus SNP pairs over the preliminary set (unfiltered HFCC results).

A total of 36 SNPs were identified as tracking SNPs in the two methods using tracking filter.

#### Noise filters (control, direction, tracking)

##### Direction filter

When the control filter is not used, the direction filter has a large impact on the number of results selected. Of the 418,535 preliminary associations, only 76.5 % (320,265) had effects in the same direction in all replication groups. These results can then be grouped into those with marginal effects in both loci (simultaneous), only one marginal effect (conditional), and no marginal effect (epistatic). A marginal effect was defined as a single-locus effect with a chi-squared (1 df) statistic larger than 3.84 (p-value < .05). This liberal cut-off serves our goal of selecting as pure epistatic effects those marker combinations with no or small marginal effects. Under these criteria, 22.3% (71,332) of the results had simultaneous marginal effects, 77.7% (248,898) had conditional effects, and only 0.01% (35) had epistatic effects. At this point, out of the hundreds of thousands of preliminary results, only 35 two-locus associations without marginal effects remained, very likely to be epistatic interactions. The many thousand simultaneous and conditional results may also involve epistatic effects on top of the marginal effects, but the large number of them prevents a thorough post-hoc analysis.

##### Direction and Tracking filters

In order to distil further the post-direction-filter results we can apply the tracking filter. Many of these two-locus associations are due to what we have denominated tracking loci, that is, markers with large marginal effects, which therefore display significant two-locus effects with many other markers. Most of these tracking markers exhibit large association effects because they fail HWE, and they are filtered out at this stage. In this Parkinson dataset we detected 36 tracking markers, defined as those markers showing up in 270 or more two-locus associations (top 0.1% of post-direction-filter results). Most of these tracking markers (83.3%) failed HWE (p < 0.001 in controls or <0.0001 in cases), and others failed minor allele frequency (MAF<0.1) or call rate (CR<90%) criteria. Therefore, results involving these markers can be safely excluded.

A few of these tracking markers represent the best single-locus association results which, due to their large marginal effects, also appear in a large number of two-locus association results. These large-single-locus-effect tracking markers need their own specific post-hoc analysis. Because of their large marginal effects, these markers are likely identifiable by single-locus analysis, but it is still noteworthy to discover if they have epistatic effects, and with which genes. However, it is hard to identify a significant epistatic interaction in the background of such a large marginal effect. Individual post-hoc analysis for each of these markers may identify the most likely multi-locus combination involving those marker.

Interestingly, by filtering out the marker combinations involving the tracking markers, we can eliminate 91.8% of the previous positive results. Most of these results were conditional effects, involving the tracking locus (with a large single-locus effect) and a tracked locus (with no main effect, but tracked by the large effect in the other locus). The post-tracking-filter results include a total of 35 marker variables with epistatic effects, 6,706 with conditional effects, and 19,630 with simultaneous effects.

##### Control, Direction and Tracking filters

We can repeat this data filtering process, but this time starting by applying the control noise filter. The control filter was set to employ the same statistical cut-off (chi-squared = 6.64) used in the case-control comparisons. Association results above this cut-off value obtained in analysis of two control groups can be deemed problematic and excluded from further analysis. This control filter was able to eliminate 18.75% of the preliminary results, leaving a total of 340,043 associations (81.25%). Applying the direction filter to the post-control-filter results eliminates only a further 5.84% of preliminary results, yielding a total of 320,188 associations, almost exactly the same quantity as when the direction filter was used alone. Therefore, the control and direction filters seem to eliminate most of the same results, which reassures their correct functioning and also allow for different analysis strategies. On one hand, when enough control subjects are not available and consequently the control filter can not be used, these results suggest that the direction filter may work well alone. On the other hand, the direction filter may not be useful for some multiple-phenotype studies, expecting effects on different directions in different replication groups. Thus, in some scenarios, the control and tracking filters may provide enough noise elimination by themselves.

When the tracking filter was applied to the results selected by the control and direction filters, we obtained 35 two-locus combinations with epistatic effects, 6,701 with conditional effects, and 19,611 with simultaneous effects. By applying three consecutive data filters (control, direction and tracking), we excluded almost 94% of the preliminary results. The remaining 6.3% of results are subsequently analyzed for departures of additive or multiplicative two-locus models, to estimate their potential for epistatic effects.

#### Epistatic interaction indices

HFCC analysis yielded 35 two-locus combinations with epistatic effects and no noticeable single-locus marginal effects, ten of which are displayed in Table 3. In addition, there are 26,312 conditional or simultaneous two-locus combinations, which may display interaction effects over and above the marginal effects. There are a variety of tests and indices to detect departures from additive or multiplicative models. For example, a case-only chi-squared test can detect two-locus interactions which deviate from a multiplicative model. For the present study, this case-only statistic was used to choose, among the conditional and simultaneous marker variables, those with the most significant interactions (Table 3). In addition, marker quality control criteria were checked to assure all markers in selected combinations passed a minimum requirement (HWE p-value > 0.001 in controls and >0.0001 in cases; MAF>0.1; call rate>90%).

**Table 3:** Selected Parkinson's disease two-locus combinations.

SNP1	SNP2	Effect	Genetic Model	Odds Ratio	2-locus X^2^	Case-only X^2^
rs6542522	rs3923511	E	TC*GA	0.28	24.97	13.07
rs10799573	rs1341622	E	CC*CT	5.22	23.08	10.61
rs12520264	rs2992630	E	AG*GA	0.22	23.86	23.12
rs324454	rs12672177	E	GG*AG	0.21	24.31	11.55
rs6656554	rs3898966	E	GT*TC	3.83	23.44	22.34
rs7650598	rs12353255	E	AG*AG	3.99	23.94	9.40
rs2439525	rs1327918	E	TT*CC	2.39	22.04	7.13
rs10201616	rs4495512	E	CT*TT	5.04	24.08	8.63
rs11167062	rs1270919	E	TC*GT	0.20	21.83	20.65
rs2419117	rs10512174	E	GT*CC	5.55	23.33	9.33
rs1370699	rs1673130	C	CC*CC	0.35	25.50	30.15
rs1590957	rs8043401	C	TT*AG	0.32	24.17	28.18
rs1557615	rs2582597	C	TC*AG	0.35	21.76	26.38
rs11781101	rs4775501	C	CC*CC	0.22	23.99	25.69
rs1370699	rs7897163	C	CC*CC	0.34	30.65	25.68
rs13197142	rs2206699	C	GG*AA	2.33	21.85	25.16
rs732594	rs12373417	C	AA*GG	3.62	22.31	25.16
rs10498269	rs1551355	C	TC*CC	0.38	23.42	25.00
rs2555614	rs331617	C	AA*TT	0.35	24.15	24.22
rs3894377	rs10774863	C	AG*CT	0.31	25.47	23.84
rs4799327	rs2301661	S	AG*CC	4.32	20.65	33.62
rs2336865	rs7973385	S	GA*TC	3.22	30.71	27.77
rs6779648	rs270406	S	GG*AG	0.26	26.57	22.49
rs12599027	rs767055	S	TT*TT	0.23	26.23	24.34
rs3891371	rs4724620	S	AG*CC	0.29	35.76	23.92
rs2297518	rs660454	S	AG*TT	0.12	23.92	23.72
rs357968	rs1159145	S	AA*AA	0.26	30.29	23.47
rs1476097	rs5766305	S	TT*TT	0.37	23.93	23.19
rs2955005	rs2169793	S	GT*AG	3.29	25.74	22.76
rs2560790	rs9390939	S	AA*CA	0.32	23.35	22.62

A selection of two-locus marker combinations consistently and significantly associated to Parkinson's disease (p < 10^-6^) and displaying an epistatic (E) effect, or conditional (C) or simultaneous (S) effects with significant deviations of a multiplicative model. The genetic model tested is specified as SNP1 genotype * SNP2 genotype.

#### Validation analysis

In order to validate to some extent these results, and without immediate access to a truly independent sample, we created 10 validation samples by randomly re-creating the case groups (F) from the full pool of cases (N = 270), and similarly for the control groups (C). In the described analysis of the Parkinson dataset, three replication groups were created randomly, to find consistent results across these groups. To examine the possible effect of this random group membership, the replication groups were re-created randomly and reanalysed in what we called validation experiments. These validation experiments are not permutation simulations to estimate the empirical significance level of parameter estimates, and are not a standard bootstrapping technique because they use sampling without replacement. The replication groups in each validation analysis were re-created under association (keeping cases as cases and controls as controls), and not under the null hypothesis of no association. These validation experiments are used to examine the consistency of results when the replication groups are re-created.

The control noise filter was not employed for these validations analyses to save computing time, since it was found that for this dataset the direction and tracking filters alone were sufficiently efficient. Because we are using the same set of cases and controls in all the validation groups, a high degree of consistency of results across validations analyses would be expected a priori. Nonetheless, due to the small sample size of each group, the many requirements for the selection of marker variables, such as the consistency of strength and direction of result in three replication groups, and the subjective categorization (single-locus X^2 ^> 3.84) of results into different types of effects (epistatic, conditional and simultaneous), a large variability in results across validation analyses is reasonable.

The total number of preliminary results varies noticeably across validation groups (Table 4). This variability illustrates the randomness inherent in this type of analysis. The total number of two-locus combinations selected as simultaneous or conditional are much more consistent. Nonetheless, the number of epistatic effects is quite variable again, perhaps a consequence of the small number of marker combinations selected under this category.

**Table 4:** Validation analysis.

Sample	Preliminary Results	Simultaneous	Conditional	Epistatic
Original	418,535	19,630	6,706	35
Validation 1	396,708	20,855	6,897	23
Validation 2	283,584	19,270	6,219	18
Validation 3	299,846	19,261	6,000	27
Validation 4	403,475	19,640	6,660	21
Validation 5	337,422	19,575	6,463	22
Validation 6	320,406	18,879	6,230	20
Validation 7	313,653	18,667	5,943	21
Validation 8	294,007	19,255	6,360	26
Validation 9	322,754	18,478	5,772	22
Validation 10	363,194	19,317	5,529	28
Average Count (St. Dev.)	341,235 (47,125)	19,348 (629)	6,253 (419)	24 (5)
Average %	100%	7.92%	2.56%	0.010%

Number of total preliminary results, and number of selected simultaneous, conditional and epistatic two-locus combinations in the original sample, and in ten random-placement validation samples. Average numbers (and standard deviations) for the 11 samples, and average percentage of each category, are included.

In addition to the variability in the number of results, it is important to note the general lack of consistent results across validation groups. The simultaneous effects were the most consistent type of result. One two-locus combination (rs7653784 rs499091) showed up in the original analysis and the 10 validation samples. Five other combinations with simultaneous effects were consistent in 10 out of the 11 samples, and 28 combinations were consistent in 9 samples. Of the conditional results, thirteen marker combinations were consistent in 8 samples. Perhaps because their effect is harder to replicate, only one epistatic marker combination was present in as much as 5 samples, while 2 combinations were present in 4 samples, and three combinations in three samples.

## Discussion

HFCC is a new computer software for exhaustive genome-wide analysis of multi-locus association effects in a case-control design. It carries out different types of statistical tests to assess a variety of genetic and epistatic models. HFCC differs from other association or multi-locus methods in that it can analyze simultaneously multiple samples or multiple phenotypes, and incorporates several complementary noise-signal filters, and also post-hoc analysis tools. To address the enormous computing task, it is elegantly designed to take advantage of the multiple CPUs available nowadays in computer servers or clusters.

The goal of HFCC analysis is to find multi-locus marker combinations which are significantly associated with a phenotype, especially those displaying interaction effects which may not be detected in a single-locus analysis. By setting the type of genetic model, the number of subjects in each group, the number of replication groups, the statistical cut-offs for the different tests, and applying different noise elimination filters, HFCC can arrive at a selection of the most promising multi-locus combinations.

Other multi-locus methods to detect gene-gene interactions exist. For example MDR [10] is a method for exhaustive search of high-level multi-locus interactions, although it is extremely computationally intensive, and genome-wide searches for epistasis are prohibitive. More recently a promising Bayesian method (BEAM) has been suggested as a powerful alternative for detecting epistatic interactions, although it is not exhaustive and still requires further improvements to effectively handle the large SNP datasets commonly used in genome-wide studies [11]. Other methods, like PLINK [9] or others based on logistic regression [5], can carry out genome-wide epistasis tests relatively quick, but are currently limited to two- or three-locus models, and only perform general tests of epistasis. HFCC combines a relatively fast computing algorithm for genome-wide epistasis detection, with the flexibility to test a variety of different epistatic models in multi-locus combinations. Our analysis of a simulated dataset reveals that HFCC has good power, at least as good as or better than MDR [20], to detect epistatic interactions, as long as they are relatively strong and common. In the most extreme simulation, with 5% genotyping error, 5% missing data, 50% phenocopies, and 50% genetic heterogeneity, HFCC still had 71% power to detect some types of epistasis, although the power for other types of epistasis was smaller (34–51%). We will need to carry out more extensive simulations to evaluate the power under different conditions and genetic models.

For this illustrative application of the software we have also analyzed an open-access dataset of Parkinson's disease patients and unaffected controls. We would like to emphasize here the importance of these public datasets of real data to improve the quality of applied research, and also to foster the development of new methodology.

One of the pecularities of HFCC is the possibility of dividing the case-control sample into replication groups, to detect only those effects that are consistent across samples. The number of replication case-control groups to be used depends on the available dataset (sample size and number of genetic markers). It is an important analysis parameter because the overall significance level is a function of the selected critical value for the test statistic and the number of replication groups. For the current analysis, the sample of cases was divided into three replication groups, and so were the controls. This strategy focuses on the detection of large and consistent effects, which are hopefully detectable with the available datasets. It is reasonable to expect that the joint effect of a combination of genes is larger and more penetrant than each of the single-locus effects, and therefore under some circumstances (i.e., not extremely heterogeneous or rare effect), these multi-locus effects can be identified. The detection of small, rare or heterogeneous effects may need larger samples and more complex models.

HFCC allows for a variety of different genetic models and tests. Different models may be necessary to detect different types of effects, such as recessive, dominant or heterozygote effects. The best analysis strategy may depend on prior knowledge or hypothesis about the trait. An optimal strategy would apply a selected subset of models which would maximize the chances of detecting a hypothesized effect. For example, for this Parkinson's disease study, we have employed a subset of nine epistatic models which typically detects recessive effects.

Another characteristic of HFCC analysis is the successive application of noise-signal filters. Control groups can be compared against each other to remove background noise associations. Direction of effect can be taken into account, so that only those results consistent in strength and direction across replication samples are selected. A final filter is able to remove those multi-locus results which are primarily due to quality-control failing markers (ie, in Hardy-Weinberg disequilibrium, low allele frequency or low call rate) or to large single-locus effects. The remaining multi-locus combinations can be categorized into epistatic, conditional and simultaneous effects, and interaction tests can be used to detect possible epistatic interactions over and above the marginal effects. The selected markers can then be included in a validation analysis in an independent sample. As an illustration, Table 4 displays 30 two-locus combinations suggestive of displaying epistatic interactions influencing the development of Parkinson's disease. It is important to note that due to the small sample size used in this experiment, these results may not be reliable, and need re-analysis or confirmation in larger datasets. The number of combinations selected for a validation analysis depends on many factors, and tools are included to help perform this selection.

The Parkinson's disease study reported here can illustrate several issues regarding the search for epistatic effects in large datasets. One of the most difficult tasks in large dataset analysis is selecting the most promising candidate results. The huge number of statistical tests performed requires a severe statistical cut-off, or a protocol of data filtering, to be able to select only the most promising results. For example, the two-locus analysis of a genome-wide association SNP dataset presented here reveals several hundred thousands two-locus marker combinations at a liberal significance level (i.e., p value < 10^-6^). Using a stringer significance level, such as a Bonferroni correction, may be overly conservative, sometimes potentially missing real effects. HFCC filters and post-hoc analyses help selecting the most promising two-locus interactions from a large set of preliminary findings. For example, all two-marker combinations in Table 4 are consistently associated with Parkison's disease in three replication groups (overall two-locus X^2 ^(1 df) in the range 21–30), and they all also deviate from a multiplicative model (case-only X^2 ^(1 df) in the range 7–33) suggesting an epistatic interaction.

The resampling validation analysis raises an important issue regarding the difficulty in replicating a result across different validation samples, a finding that may reflect the general lack of power to detect these types of effects, especially in the presence of heterogeneity. With the available sample size for this study we have approximately 80% power to detect large common effects (Odds ratio > 3 in a genotype prevalence > 25%) at a significance level of 0.01 per replication group. However, this small dataset is underpowered to find more moderate, and perhaps more realistic, effect sizes. The general lack of consistency suggested by our own sensitivity analysis may be a consequence of the small sample size analyzed. Fung et al. (2006) claimed that there is no common genetic variant that exerts a large genetic risk for late-onset Parkinson's disease in white North Americans. Multi-locus analysis may, however, reveal the existence of large complex (multi-locus) genetic effects.

### Analysis Guidelines

HFCC provides a tool for the genome-wide study of epistasis. Its use may depend heavily on the researcher's goals and the data available. For this reason, it is hard to provide general guidelines on the optimal parameters for analysis, but HFCC's flexibility to accommodate to the specific needs of each study is a great asset.

A key parameter is the number of replication groups. When the available sample size is fixed, dividing the sample into more replication groups decreases the power of the analysis, but also increases the confidence in the remaining results. For example, the two-locus analysis of the full Parkinson's dataset in one case-control group with a Type I error set at 10^-6 ^yields a total of 784,506 preliminary positive results. The analysis of the same data splitted in three replication groups, each with alpha = 10^-2^, yields only 418,535 results (53% of the single-group results).

There is not an optimal number of replication groups. Researchers need to select this parameter as well as the significance level cut-off to fit their dataset and study design. For example, a study of three related diseases or phenotypes suggests the use of three replication groups. In the case of a single disease, the number of replication groups, the sample size in each group, and the statistical cut-off should be chosen depending on the nature of the study. A strict statistical correction may be necessary if the results are to be conclusive, while a more relaxed criterion may be used in a two-stage study where the goal of the preliminary analysis is to select a subset of markers for subsequent validation.

HFCC's flexibility is also possible in the application of data filters. The results displayed in Table 2 suggest that the control filter and the direction filter can eliminate most of the same noise results. The application of either or both of these filters can therefore depend on the study design. If a study has many control subjects and a limited number of cases, it can benefit from using the control filter. If a study has many affected individuals, then several replication groups and the direction filter can be used.

There are also 255 possible two-marker genetic models that can be tested. Many of them are correlated, so it is probably not necessary to test them all. We suggest using a small subsample of models that cover the researcher's hypothesis. For example, for the Parkinson's analysis in this paper we tested nine simple genetic models. To identify subsamples of models that may optimize the chances of discovering epistatic effects of different nature would need a thorough simulation analysis that is beyond the scope of the current paper. Another analysis option is to use more general tests of epistasis, which are also implemented in the software.

### Statistical Issues

The two-locus analysis of the full Parkinson's dataset as presented here comprises a total of 708 × 10^9 ^statistical tests. The two-locus analysis of these data in one case-control group with a Type I error set at 10^-6 ^yields a total of 784,506 preliminary positive results, about 10% more of those expected by chance. This false positive inflation is probably due to the tracking markers (mainly QC-fail markers) as well as to the correlated nature of some of the statistical tests, and can be controlled by the use of replication groups, or by applying more stringent cut-offs if necessary.

For the simulated dataset, analyzed with only one case-control group a significance level of 10^-2 ^(chi-square 6.64), the Type I error was actually lower than expected (approximately around 0.006 on average). These results demonstrate that HFCC analysis is not only powerful but can also be conservative, preserving against Types I and II Errors. More thorough simulations to assess the impact of sample size, allele frequency, number of replication groups, and noise filter applications are needed in the future to understand these issues in detail.

Applying strict multiple testing correction (Bonferroni) to the Parkison's disease analysis, we do not find any significant two-locus interactions. The reason for this may be the small sample size available in the Parkinson's dataset. But we can still select the most promising two-locus results for subsequent validation. The key issue is whether we are concerned with achieving an absolute level of statistical significance, which may not be properly defined in this setting given the complexity of the analysis suggested (multiple testing of correlated hypotheses, independent replication of results, data filtering steps), or selecting the most promising markers or marker combinations that pass a more or less stringent statistical criterion. In either case, the usefulness of HFCC for selecting marker combinations for later validation is doubtless.

### Computational Issues

Multi-locus analysis is computationally intensive and is therefore limited by computing capabilities. HFCC is a relatively fast algorithm considering the huge number of computations it performs. The dataset analyzed in this study consists of 396,591 genetic markers, which results in 78.6 × 10^9 ^two-locus marker combinations. Nine genetic models were tested in the current study, resulting in a total of 708 × 10^9 ^statistical tests. Moreover, these tests may be carried out in as many as three case-control replication groups, and also in as many as three control-control noise-filter groups, so the computing task is staggering. HFCC is programmed to take advantage of computer resources by dividing the computing task into processes which can migrate to the available CPUs in a computer server or cluster. For the current analysis, we employed a computer cluster consisting of twelve 3.2 GHz CPUs, which was able to carry out the full genome-wide analysis in approximately 5 days.

Another computational complication for multi-locus analysis is related to RAM memory. The data matrices need to be loaded onto memory to speed computations, and therefore a limitation exists for the analysis of very large datasets (millions of genetic markers or several thousands of subjects) where RAM memory is rapidly exhausted. This limitation, however, can be solved with parallel processing techniques (MPI like), such as dividing the data matrices into smaller subsets which are distributed around the computer network.

HFCC can perform more complex multi-locus analysis (3-locus, 4-locus, etc.), but the number of computations grows exponentially with the number of interacting markers, and the analysis becomes dependent on computing resources and time limitations. Depending on these resources, genome-wide three- or four-locus analysis may require a two-stage strategy, where some markers are selected first by single-locus analysis, and then employed to guide the multi-locus testing [19]. Our exhaustive two-locus genome-wide analysis of a Parkinson's disease dataset reveals that pure epistatic effects, as defined here, are rare (0.01% of the preliminary results). Therefore, a two-stage strategy for multi-locus analysis may be a more economical analysis with minimal information loss. This statement assumes that we had power to detect these epistatic effects, that more complex interactions behave as two-locus ones, and that Parkinson's disease is representative of other diseases. Our results suggest the use of a conditional two-stage strategy, where a liberal single-locus threshold is first used to select loci with marginal effects, and then these markers are used against the full panel for multi-locus analysis. This conclusion is similar to some previous suggestions [5,19] but not all [21], confirming that a liberal single-locus cut-off (i.e., p < .05) greatly reduces the computational task while minimizing the probability of discarding potential epistatic loci.

It is also important to note that linkage disequilibrium (LD) is unaccounted for in our analyses. LD reflects an association among markers and therefore can affect the results of some tests. For example, it can produce a significant case-only chi-squared test. Nonetheless, HFCC's algorithm and analysis filters seem to prevent this bias. In the case where one marker is associated with several markers in LD, these results are detected in the last stage of marker selection.

## Conclusion

In summary, we propose that genome-wide multi-locus analysis is performed on available datasets of common diseases, because they can exploit the large genetic datasets and computing resources becoming available, to open a new phase of genetic analysis. The analysis of Parkinson's disease reported here represents the first exhaustive genome-wide epistasis search on a real dataset, effectively handling hundred of thousands of genetic markers, and demonstrating its feasibility. Due to the small sample size, however, these results are only illustrative and require re-analysis or confirmation in larger datasets. These multi-locus analyses would not substitute conventional single-locus analysis but add a new layer of genome-wide association studies, allowing the identification of new candidate markers for further validation. HFCC is a new genome-wide multi-locus software, which allows the user a high degree of control over analysis parameters, so that data analysis can be tailored to the specific needs of each project. HFCC can have a great impact on the discovery of the genetic causes of common diseases, especially to identify those multi-locus effects that may not be detectable using the available single-locus methods. The discovery of new genes affecting a disease may be useful as predictive tools or to find new therapeutic targets. HFCC has multiple applications, not only in the study of disease phenotypes, but also of other qualitative traits, and can be used in clinical trials or pharmacogenetics studies.

## Availability and requirements

HFCC is written in C and freely available for linux platforms from this Website: 

## List of abbreviations

CPU: central processing unit; GE: genotyping error; GH: genotyping heterogeneity; HFCC: hypothesis free clinical cloning; HWE: Hardy-Weinberg equilibrium; LD: linkage disequilibrium; MS: missing data; PC: phenocopy; SNP: single nucleotide polymorphism; XOR: logical exclusive "or" statement.

## Authors' contributions

JG, AG–P, and AR conceived and designed the experiments. JG, AG–P and AR analyzed the data. JG and AR wrote the paper. AR conceived the analysis tool. All authors helped develop the analysis tool. FB and AQ wrote the analysis tool. All authors read and approved the final manuscript.

## Competing Interests

As a declaration of competing financial interest, authors in the paper are employees and/or shareholders in Neocodex. Neocodex owns a patent on  the HFCC algorithm described in this paper (WO 2008010195 20080124).
